# Bioanalytical Method Development and Validation of Veratraldehyde and Its Metabolite Veratric Acid in Rat Plasma: An Application for a Pharmacokinetic Study

**DOI:** 10.3390/molecules25122800

**Published:** 2020-06-17

**Authors:** Hyun Wook Huh, Hee-Yong Song, Young-Guk Na, Minki Kim, Mingu Han, Thi Mai Anh Pham, Hyeonmin Lee, Jungkyu Suh, Seok-Jong Lee, Hong-Ki Lee, Cheong-Weon Cho

**Affiliations:** 1 College of Pharmacy, Chungnam National University, Daejeon 34134, Korea; hhw3573@nate.com (H.W.H.); song314@kup.co.kr (H.-Y.S.); youngguk@cnu.ac.kr (Y.-G.N.); zkzkang@naver.com (M.K.); linuxfalcon@naver.com (M.H.); phammaianhdkh68@gmail.com (T.M.A.P.); gusals2218@naver.com (H.L.); 2 Woojung Bio Inc., 145, Gwanggyo-ro, Yeongtong-gu, Suwon-si 16229, Korea; jksuh@woojungbio.kr (J.S.); seokjonglee@woojungbio.kr (S.-J.L.)

**Keywords:** veratraldehyde, veratric acid, bioanalytical method, UHPLC, triple quadrupole mass spectrometer, pharmacokinetics

## Abstract

A simple, sensitive, and rapid UHPLC-MS/MS method was developed for the simultaneous determination of veratraldehyde and its metabolite veratric acid in rat plasma. Cinnamaldehyde was used as an internal standard (IS) and the one-step protein precipitation method with 0.2% formic acid in acetonitrile (mobile phase B) was used for the sample extraction. Reversed C18 column (YMC-Triart C18 column, 50 mm × 2.0 mm, 1.9 µm) was used for chromatographic separation and was maintained at 30 °C. The total run time was 4.5 min and the electrospray ionization in positive mode was used with the transition *m/z* 167.07 → 139.00 for veratraldehyde, *m/z* 183.07 → 139.00 for veratric acid, and *m/z* 133.00 → 55.00 for IS. The developed method exhibited good linearity (*r*^2^  ≥  0.9977), and the lower limits of quantification ranged from 3 to 10 ng/mL for the two analytes. Intra-day precision and accuracy parameters met the criteria (within ±15%) during the validation. The bioanalytical method was applied for the determination of veratraldehyde and veratric acid in rat plasma after oral and percutaneous administration of 300 and 600 mg/kg veratraldehyde. Using the analytical methods established in this study, we can confirm the absorption and metabolism of veratraldehyde in rats for various routes.

## 1. Introduction

Veratraldehyde (3,4-dimethoxybenzaldehyde, VD), a derivative of vanillin, is the chemical that is found and isolated from peppermint, ginger, bourbon vanilla, and fruits such as raspberry [1]. VD has the vanilla-like and woody savor, so it is widely used as a valuable flavoring and fragrance ingredient [2,3]. In the field of chemistry, the synthesis method of VD has been comprehensively studied and there are many attempts at a catalytic green process, which is an eco-friendly catalytic process, in order to acquire the highest yield [4,5,6]. Furthermore, VD is widely used in the pharmaceutical industry as the intermediate for organic synthesis [7,8,9,10]. Moreover, there is an attempt to apply VD as a temporary consolidant [11,12]. Interestingly, some research showed the repellent effect of VD [13]. In fact, essential oil, especially peppermint oil, was well known for strong mosquito repellent action and its action has been widely reported [14,15,16]. Thus, it is known that VD plays an important role in the mosquito repellent effect of peppermint [13]. There are some attempts to use VD for one of the repellent compositions. Considering the intended use of VD, VD could directly be exposed to a human. This means that exposure to VD may be associated with its toxicity to humans. Moreover, it has been reported that VD acts as a redox cycle agent [17].

**Figure 1:**
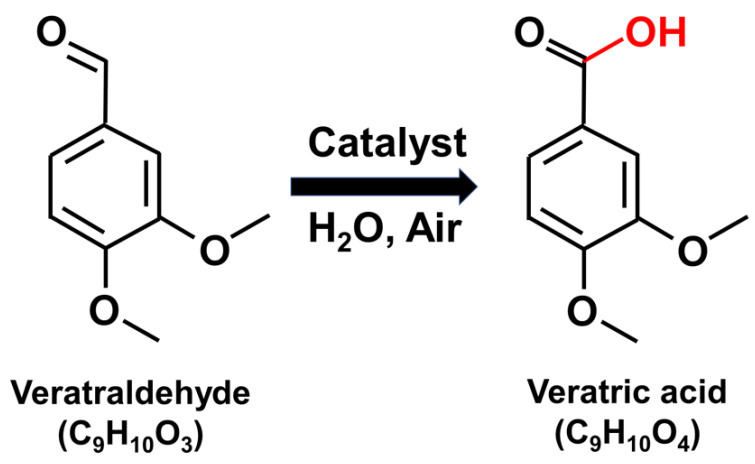
Catalytic oxidation of veratraldehyde to veratric acid.

Therefore, it is necessary to identify VD and its metabolites in biomatrix samples to assess the exposure of veratric acid (VA). It is reported that VD is metabolized to veratryl alcohol, isovanillyl alcohol, 4-methylcatechol, protocatechuic acid, catechol, and mainly veratric acid (3,4-dimethoxybenzoic acid, VA) by the intestinal microflora [18]. However, there is a lack of information about the fate of VD. According to Sammons et al. (1945), about 70% of VD was mainly metabolized to VA via catalytic oxidation (Figure 1) in rabbits [19]. Moreover, to the author’s knowledge, there is no study of pharmacokinetics or toxicokinetics of VD. Only the pharmacokinetic study and bioanalytic method for VA have been reported in rats and human plasma, respectively [20,21]. Thus, the bioanalytic method for both VD and VA should be established for the assessment of VD exposure. In addition, the evaluation of pharmacokinetics of VD would be the foundation for future toxicological or regulatory study.

For the simultaneous determination of VD and VA in rat plasma, a simple, sensitive, and rapid UHPLC-MS/MS method was developed in this study. To date, there has been no research on the development of assays for the determination of plasma concentrations of VD and its metabolites and their application to pharmacokinetic studies. It is anticipated that this study would provide the fundamental information of VD behavior for future toxicological or pharmacological studies.

## 2. Results and Discussion

### 2.1. Bioanalytical Method Development

#### 2.1.1. Determination of Mass Spectrometry Conditions

**Figure 2:**
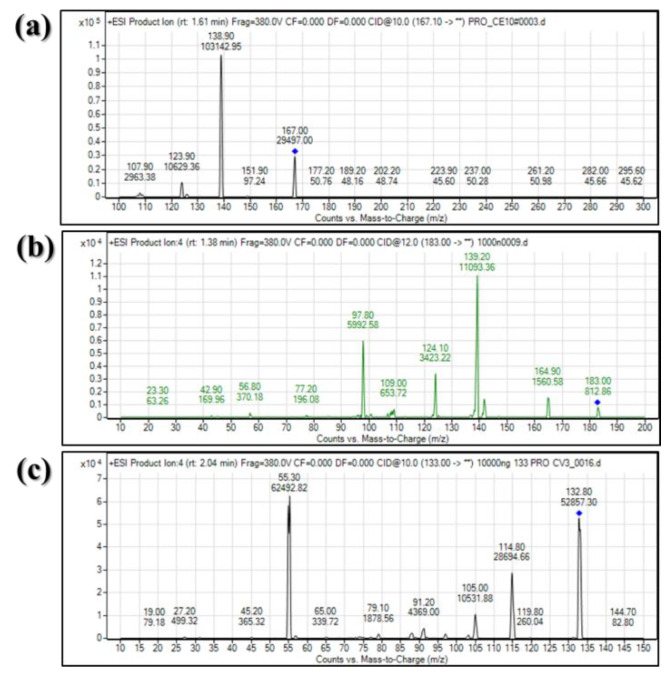
Collision-induced dissociation mass spectrum of veratraldehyde (1 µg/mL) (**a**), veratric acid (1 µg/mL) (**b**), and cinnamaldehyde (internal standard (IS) 10 µg/mL) (**c**) in positive ion electrospray ionization mode.

For the optimization of mass spectrometry conditions, both positive and negative ESI modes were investigated. The VD, VA, and internal standard (IS) showed a strong response in the positive mode (Figure 2). The fragment ion for multiple reaction-monitoring (MRM) transition was selected based on MS spectrum intensity. Parent ions for VD, VA, and IS (*m/z* 167.07, 183.07, and 133.00, respectively) were fragmented at *m/z* 139.00, 139.00, and 55, respectively (Table 1).

#### 2.1.2. Determination of Liquid Chromatography Conditions

The liquid chromatography condition and mass spectrometry condition were developed in parallel. Isocratic elution was conducted with various combination of mobile phase (methanol–distilled water (DW) or acetonitrile (ACN)-DW). Mobile phase with ACN-DW showed better signal intensity of VD and was selected for further experiments. Formic acid (0.2%, *v/v*) was used for both mobile phases A and B to induce sufficient ionization of the analytes and reduces fronting and tailing of VD. Double peaks occurred when an injection volume of 5 µL was attempted, and a sharp peak was obtained after reducing to 2 µL. A flow rate of 0.3 mL/min was also used to control the retention time of the IS. The optimized total run time was 4.5 min.

#### 2.1.3. Determination of Biological Matrix and Extraction Procedure

A one-step protein precipitation method using various organic solvents was carried out for sample preparation [22]. When the protein was precipitated using methanol, tailing occurred at the peak of IS. Meanwhile, when the protein was precipitated using only ACN, sufficient quantitation could not be obtained owing to the matrix effect of VA at low concentrations. Finally, the most optimal analytical results were obtained when protein precipitation was carried out using 300 µL of 0.2% formic acid in ACN (mobile phase B). The optimized extraction method was simple and showed the highest recovery for all analytes. The recovery values of VD and VA ranged from 99.44% to 104.14% and from 94.65% to 104.20%, respectively.

### 2.2. Bioanalytic Method Validation

#### 2.2.1. Linearity and Lower Limit of Quantification (LLOQ)

In accordance with the European Medicines Agency (EMA) guidelines, the independent LLOQ and quality control (QC) samples were used and analyzed in this study. The bioanalytical method showed the linear relationship over the range of 3–1000 ng/mL and 10–10,000 ng/mL for VD and VA, respectively. In addition, the assessed correlation coefficients (*r*^2^) were >0.99 for both VD and VA. The LLOQ was selected as five times the signal-to-noise (S/N) with acceptable accuracy and precision values (±15%). Table 2 listed the regression equations, linear ranges, and LLOQs.

#### 2.2.2. Specificity

**Figure 3:**
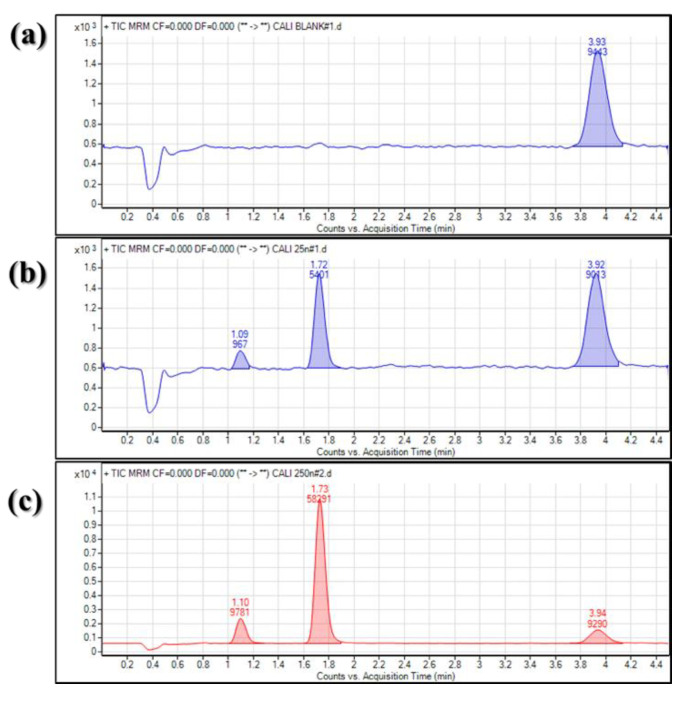
Representative multiple reaction-monitoring (MRM) chromatograms of blank plasma (**a**); blank plasma spiked with internal standard (IS) (**b**); 25 ng/mL plasma sample (**c**); and 250 ng/mL plasma sample.

To evaluate the specificity of bioanalytical method, the signal stability, response, and carryover of analytes were assessed. In this analytical method, ion suppression occurred when the analyte responses between in solvent and matrix were compared. However, chromatographic separation was well performed, so it did not affect the quantitative and qualitative analysis of VD and VA (Figure 3). When the retention time of analytes and IS was evaluated, the coefficient of variation (CV) value did not exceed 0.5%. In addition, the CV value of each analyte/IS peak area ratio was below 7.0%.

#### 2.2.3. Precision and Accuracy, Matrix Effect, and Carry Over

The intra- and inter-day precision (% relative standard deviation (RSD)) were less by 4.11% and 7.50%, respectively, for both of the analytes (Table 3). The intra- and inter-day accuracy (relative error (RE)) was found to be within ±11.57% for all of the analytes and QC samples (Table 3). Matrix effect data for VD, VA, and IS at the two QC levels are calculated. Matrix effect precision for each analyte was less than 7.84%, indicating the matrix effect of the bioanalytical method was acceptable. Area of blank plasma samples compared with the area of LLOQ and IS was not quantifiable for the calculation. Moreover, no significant carry over was observed after the sample injection. This means that no carry over of blank plasma samples was observed.

#### 2.2.4. Stability

In the stability test, VD and VA showed acceptable stability in rat plasma at room temperature and autosampler for at least 24 h, −80 °C for 4 weeks, and three freeze–thaw cycles (Table 4). Moreover, when the stock solutions were stored at room temperature and 1 week at 4 °C, they were stable up to 24 h. The results are presented in Table 4. In the stability tests of VD and VA, RSD values were less than 5% and 15%, respectively, for all conditions. In addition, RE values for VD and VA were less 20% and 17%, respectively. In all stability test conditions, the REs for VD and VA were within ±15% except for the VD in freeze–thaw for three cycles. Although the freeze–thaw stability showed the difference of 19%, the validity of the bioanalytical method is still simple and effective for the VD and VA analysis. VD and VA were most vulnerable to freezing–thawing and post-preparation processes, respectively.

### 2.3. Pharmacokinetic Application

**Figure 4:**
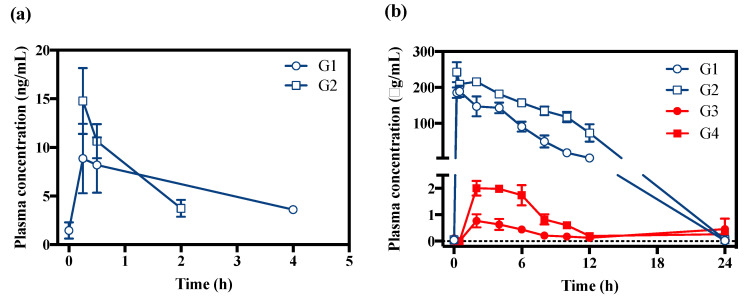
Plasma concentration versus time profiles for veratraldehyde (**a**) and veratric acid (**b**) after oral (G1, 300 mg/kg; G2, 600 mg/kg) and percutaneous (G3, 300 mg/kg; G4, 600 mg/kg) administration of veratraldehyde (*n* = 3).

The bioanalytical method developed in this study was successfully applied to determine VD and VA in rat plasma samples after oral and percutaneous administration of VD. The blood concentration–time curves for both analytes are shown in Figure 4. Pharmacokinetic parameters are listed in Table 5. Pharmacokinetic data for VD and its metabolite VA have not been previously reported. After the percutaneous administration of VD, the VD concentration was measured below the LLOQ. VD was detectable in the plasma up to 2 and 4 h for G1 and G2 group, respectively (Figure 4a), while the large amount of VA was detected for up to 24 h in the oral and percutaneous group, respectively (Figure 4b). As per our current result, the plasma concentration of VA was much higher than that of VD regardless of dose route and amount, which implied that VD is immediately metabolized to VA. When the metabolite ratio (MR) was evaluated by dividing the VD’s area under the concentration–time curve (AUC)_0–4_ to VA’s AUC_0–4_, values of MR were 4.5 × 10^−5^ and 3.6 × 10^−5^ for oral treatment groups at 300 and 600 mg/kg, respectively (Table 5). This indicates that the VD was excessively metabolized to VA after the oral administration of VD. In the cases of both VD and VA, the plasma concentration in the oral treatment group was superior to that in the percutaneous treatment group (Figure 4). In addition, the rapid absorption of VD was observed after the oral administration of VD. To the author’s knowledge, there is only one piece of literature about the pharmacokinetics of VA [19]. According to the previous pharmacokinetic study of VA, the half-life ranged from 71 to 86 min after the intravenous injection of VA at doses of 2.5, 5, and 10 mg/kg [19]. These values are similar to those of the oral administration group. Although the VD level was lower than the VA, the VD was entered into the systemic circulation. Thus, it is necessary to evaluate both VD and VA in the body when the VD is exposed to a human. Herein, we successfully applied the bioanalytical method for both VD and VA. Only a few animals (*n* = 3) were used in this study, however, preliminary pharmacokinetic studies with the sample size of 3 have been reported [23,24,25]. Thus, further study using a large number of animals is needed to clarify the pharmacokinetic profile of VD.

## 3. Materials and Methods 

### 3.1. Chemicals and Reagents

The reference standard of VD, VA, and trans-cinnamaldehyde (IS) was purchased from Sigma-Aldrich (St. Louis, MO, USA). Formic acid (99.0%) was obtained from Samchun Pure Chemical (Pyeongtaek, Korea). An Elga Purelab Option Q purification system was used in preparation of ultrapure distilled water (DW). All the other reagents were of analytical grade and obtained from Sigma-Aldrich (St. Louis, MO, USA).

### 3.2. Bioanalytical Method

#### 3.2.1. Instrumentation and Chromatographic Conditions

An Agilent UHPLC system with triple quadrupole mass spectrometer (TQ-MS) (Agilent 1290 Infinity Series, Agilent Technologies, Waldbronn, Germany) was used. In brief, the chromatographic separation was performed using a YMC-Triart C18 column (50 mm × 2.0 mm, 1.9 μm) maintained at 30 °C. The separation was performed using an isocratic flow of a solvent consisted of 70% DW containing 0.2% formic acid and 30% acetonitrile (ACN) containing 0.2% formic acid. The optimal flow rate was set at 0.3 mL/min with a sample injection volume of 2 µL. For TQ-MS conditions, gas temperature and flow were set at 200 °C and 14 L/min, respectively. The capillary voltage and cell accelerator voltage were 3000 V and 3 V, respectively. The nebulizer pressure was 20 psi. The collision energy (CE) was optimized for each compound and a positive multiple reaction-monitoring (MRM) mode was applied. Positive MRM was used to analyze VD and VA using IS.

#### 3.2.2. Preparation of Stock Standards and Quality Controls (QCs)

Stock solution (1 mg/mL) of VD and VA was prepared by dissolving in ACN and stored at 4 °C. Working solution was prepared by serial dilution of stock solution with ACN. Working standard solution was stored at 4 °C. Stock and working solution for calibration standard (CS) and quality control (QC) samples were prepared by separate weighing of the reference compound. The CS samples used in the matrix stability study were immediately prepared before the experiment. Low, medium, and high integrity QC (LOQC, MIQC, and HIQC, respectively) samples were prepared to 10, 500, and 1000 ng/mL for VD and 30, 5000, and 10,000 ng/mL for VA, respectively. In brief, 10 µL of working solution of VD or VA (100, 5000, and 10,000 ng/mL for VD; 300, 50,000, and 100,000 ng/mL for VA) was added into 90 µL of plasma for CS and QC samples, and then they were vortexed. Stock IS solution (1 mg/mL) of cinnamaldehyde was prepared by dissolving the dry compounds in methanol. Working IS solution (10 μg/mL) was prepared by the dilution of stock IS solution with ACN and stored at 4 °C. Calibrator samples were prepared to 3, 5, 10, 25, 50, 100, 250, 500, and 1000 ng/mL for VD and 10, 25, 50, 100, 250, 500, 1000, 2500, 5000, and 10,000 ng/mL for VA, respectively. In brief, 10 µL of working solution (×10 of each calibrator) was added to 90 µL of rat plasma. Then, samples were extracted by the method described in Section 3.2.3.

#### 3.2.3. Plasma Sample Extraction

To optimize the extraction method, the various organic solutions, including MeOH, ACN, and EtOH, and the various ratio of DW/solvent, were used. Among them, the optimal solvent (0.2% formic acid in ACN) was selected based on the LOQ and linearity. Herein, the VD and VA was extracted by the liquid–liquid extraction method. Briefly, the plasma sample (100 µL) was added to 10 µL of IS working solution and vortexed for 5 s. Then, 0.3 mL of mobile phase B was added and shaken at 1000 rpm for 10 min. They were centrifuged at 15,000 rpm for 5 min at 4 °C. The clear supernatant was taken and injected into the UHPLC system.

#### 3.2.4. Validation of Bioanalytical Method

The developed bioanalytical method was partially validated in terms of linearity, lower limit of quantification (LLOQ), specificity, accuracy, precision, recovery, matrix effect, and stability according to the guidance on bioanalytical method validation issued by European Medicines Agency (EMA) guidelines [26]. The criteria and validation method were followed by the EMA guideline. However, the criterion for stability test was set to 20%. The calibration curves were described by plotting the peak area ratio of the VD or VA to IS against the nominal concentration of VD or VA and they were weighted (1/x). LLOQ was selected as the lowest concentration with acceptable linearity, accuracy, and precision. We analyzed VD or VA LOQC samples spiked in rat plasma for system suitability tests. The sample was injected five times. Precision and accuracy were expressed as the relative standard deviation (RSD) and relative error (RE), respectively. They were calculated for LOQC, MIQC and HIQC samples by the following equations.

(1) RSD=(Standard Deviation)/Mean×100

(2) RE=(Measured−Actual)/(Actual value)×100

For the inter-day accuracy and precision, six replicates of QC samples were analyzed three times over three different days. Matrix effects were evaluated by calculating the ratio of response in the samples spiked after the extraction process to the response in a standard solution using six different lots of rat plasma. For the carry over experiment, the first blank sample, HIQC sample, second blank sample, and LLQC sample were extracted using the method described in Section 2.2.3. and measured once in each order. The stability of VD and VA in rat plasma was evaluated using six replicates of the QC samples. The stability test was conducted under various conditions: (i) at room temperature for 24 h, (ii) at −70 °C for 4 weeks, (iii) after three freeze–thaw cycles, and (iv) at autosampler for 24 h.

### 3.3. Pharmacokinetic Application

#### 3.3.1. Animals

Sprague-Dawley rats (200.1–203.7 g, 6-week-old, male) were obtained from the Doo Yeol Biotech (Seoul, Korea). The rats were housed at 22 ± 2 °C and the water and feed were supplied ad libitum. Animals were acclimatized for one week before experiments. All animal experiments were conducted in accordance with the guidelines established by the animal ethics committee (approval number, WJIACUC 20191104-4-21) in Woojung Bio.

#### 3.3.2. Pharmacokinetics of VD

The bioanalytical method was applied to determine the plasma concentration levels of VD or VA and to evaluate the pharmacokinetics of VD and VA in rats that orally or percutaneously received the VD at doses of 300 and 600 mg/kg (Table 6). Blood samples were collected via fosse orbital vein at 0 (pre-dose), 0.25, 0.5, 1, 2, 4, 6, 8, 10, 12, and 24 h after VD administration. A single 100 µL aliquot blood was delivered to empty heparinized tubes immediately at each time point. Then, the blood samples were centrifuged at 4 °C, 3000 rpm for 10 min. The plasma samples were collected and stored at −20 °C till extraction. No side effects and signs of abnormality in the rats were observed during the animal studies.

## 4. Conclusions

In summary, we report the development and validation of a UHPLC-MS/MS method for the simultaneous quantification of VD and VA in rat plasma. The validated method is simple, rapid, and robust. In addition, this is the first study to develop bioanalytical method for the pharmacokinetic study of VD and VA. VD is rapidly metabolized by enzymes to the bioactive metabolite, VA. Our objective was to develop a single method capable of measurement of the absorption of VD after various routes of administration. The bioanalytical method was developed and partially validated in terms of linearity, selectivity, matrix effect, carry over, accuracy, precision, and stability. The developed bioanalytical method was simple and met the criteria of validation parameters described above. Moreover, this method was successfully applied to the pharmacokinetics of VD in oral and percutaneous administration.

## Figures and Tables

**Table 1:** Optimized mass spectrometric conditions. CE, collision energy.

Compound	Parent Ion (*m/z*, [M + H]^+^)	Product Ion (*m/z*)	Retention Time (RT) (min)
		(CE)	
Veratraldehyde	167.07	139.00 (11)	1.72
Veratric acid	183.07	139.00 (11)	1.10
Cinnamaldehyde (IS)	133.00	55.00 (11)	3.92

**Table 2:** Linearity and lower limit of quantification (LLOQ) of the LC-MS/MS method for veratraldehyde (VD) and veratric acid (VA). S/N, signal-to-noise; RSD, relative standard deviation.

**Compound**	**Linearity**						**LLOQ**					
	**Equation**		**Range (ng/mL)**		**Correlation Coefficient (*R*^2^)**		**Nominal Concentration (ng/mL)**		**Mean**	**RSD**		**S/N**
VD	Y = 0.031263x + 0.018961		3–1000		0.9986		3		2.64	9.87		15.5
VA	Y = 0.003814x + 0.008264		10–10,000		0.9977		10		10.09	12.37		8.04
**Linearity of Veratraldehyde and Veratric Acid**												
**Veratraldehyde**						**Veratric Acid**						
**Nominal Concentration (ng/mL)**		**Concentration (ng/mL)**		**Bias (%)**		**Nominal Concentration (ng/mL)**		**Concentration (ng/mL)**			**Bias (%)**	
3		2.64 ± 0.26		88.01		10		10.09 ± 1.24			100.95	
5		5.54 ± 0.22		110.83		25		28.07 ± 1.54			112.30	
10		10.16 ± 0.14		101.69		50		47.75 ± 2.11			95.51	
25		26.37 ± 0.71		105.49		100		103.36 ± 3.40			103.36	
50		47.75 ± 2.53		95.51		250		233.29 ± 0.92			93.32	
100		103.62 ± 3.06		103.62		500		476.86 ± 6.32			95.37	
250		233.87 ± 3.80		93.55		1000		952.92 ± 21.84			95.29	
500		499.98 ± 7.38		100.00		2500		2638.20 ± 248.29			105.53	
1000		1013.04 ± 23.77		101.30		5000		4891.51 ± 70.52			97.83	
						10,000		10,052.90 ± 145.56			100.53	

**Table 3:** The intra-day and inter-day precision, accuracy, and matrix effect for two analytes in rat plasma. RE, relative error.

Analytes	Nominal Concentration (ng/mL)	Intra-Day			Inter-Day			Matrix Effect	
		Calculated Concentration (ng/mL)	RSD (%)	RE (%)	Calculated Concentration (ng/mL)	RSD (%)	RE (%)	Mean	RSD
Veratraldehyde (VD)	3	3.3 ± 0.3	8.5	12.06	3.4 ± 0.3	12	11.2		
	10	10.2 ± 0.2	1.6	1.7	10.1 ± 0.8	7.50	1.44	9.3	3.07
	500	450 ± 6	1.4	−9.9	485 ± 13	2.73	−2.98	-	-
	1000	885 ± 15	1.7	−11.4	989 ± 31	3.10	−1.12	1084	4.6
Veratric acid (VA)	10	11.3 ± 0.4	12	6.7	11.7 ± 0.4	17	15.4		
	30	27.3 ± 1.1	4.1	−8.9	28.0 ± 0.9	3.18	−6.53	29.9	7.8
	5000	4422 ± 111	2.9	−11.6	4515 ± 84	1.85	−9.70	-	-
	10,000	9930 ± 372	3.7	−0.7	9921 ± 281	2.83	−0.79	9224	1.4

**Table 4:** Stability results of (**a**) veratraldehyde and (**b**) veratric acid in rat plasma (*n* = 5).

(**a**)				
**Veratraldehyde**	**Concentration (ng/mL)**		**RSD** **(%)**	**RE** **(%)**
	**Nominal**	**Calculated**		
Short-term: exposure at RT for 24 h	10	8.6 ± 0.1	1.3	13.5
	1000	1017 ± 8	0.8	1.7
Long-term: storage at −70 °C for 30 days	10	8.9 ± 0.2	2.6	10.2
	1000	990 ± 24	2.5	1.0
Freeze and thaw for three cycles: freezing at −70 °C and thawing at RT	10	8.0 ± 0.2	2.4	19.6
	1000	934 ± 22	2.3	6.7
Post-preparation: auto-sampler (4 °C) for 24 h	10	9.7 ± 0.2	2.1	3.1
	500	445 ± 6	4.7	11.1
	1000	888 ± 12	4.8	11.2
(**b**)				
**Veratric Acid**	**Concentration (ng/mL)**		**RSD** **(%)**	**RE** **(%)**
	**Nominal**	**Calculated**		
Short-term: exposure at RT for 24 h	30	29.12 ± 3.7	12.7	2.93
	10,000	9663 ± 315	3.3	3.37
Long-term: storage at −70 °C for 30 days	30	28.9 ± 2.0	6.8	3.75
	10,000	9487 ± 271	2.9	5.13
Freeze and thaw for three cycles: freezing at −70 °C and thawing at RT	30	25.9 ± 1.0	4.0	13.77
	10,000	8946 ± 39	0.4	10.54
Post-preparation: auto-sampler (4 °C) for 24 h	30	25.1 ± 0.9	3.5	16.24
	5000	4362 ± 130	3.0	12.76
	10,000	9830 ± 409	4.2	1.70

**Table 5:** Pharmacokinetic parameters of veratraldehyde and veratric acid in rats (*n* = 3).

Parameters (unit)	Veratraldehyde		Veratric Acid			
	G1	G2	G1	G2	G3	G4
T_1/2_ (h)	NA		1.39 ± 0.50	1.15 ± 0.25	14.56 ± 3.06	73.62 ± 96.06
T_max_ (h)	0.34 ± 0.15	0.25 ± 0.00	0.42 ± 0.15	0.84 ± 1.02	10.67 ± 11.55	3.34 ± 2.31
C_max_ (ng/mL)	9.18 ± 6.16	14.78 ± 5.87	192,374 ± 24,898	246,439 ± 43,352	880 ± 474	2344 ± 99
AUC_last_ (ng⋅h/mL)	12.80 ± 18.40	15.82 ± 4.79	1,092,969 ± 270,449	2,315,618 ± 333,017	14,423 ± 13,381	25,813 ± 2606
MR (VD/VA)	4.5 × 10^−5^	3.6 × 10^−5^	NA	NA	NA	NA

T_1/2_, half-life; T_max_, time to reach the maximum plasma concentration; C_max_, maximum plasma concentration; AUC_last_, observed area under the concentration–time curve; MR, metabolic rate of VD to VA; NA, not applicable.

**Table 6:** Experimental design of pharmacokinetic study.

Group	Sample	Dose	Route	Dose Volume
G1	Veratraldehyde (Low dose)	300 mg/kg	Oral	10 mL/kg
G2	Veratraldehyde (Middle dose)	600 mg/kg	Oral	10 mL/kg
G3	Veratraldehyde (Low dose)	300 mg/kg	Percutaneous	10 mL/kg
G4	Veratraldehyde (Middle dose)	600 mg/kg	Percutaneous	10 mL/kg
